# Redescription and designation of a neotype for *Caecum floridanum* (Littorinimorpha, Truncatelloidea, Caecidae) with a characterization of the protoconch and growth stages

**DOI:** 10.3897/zookeys.585.7646

**Published:** 2016-04-27

**Authors:** Silvio Felipe Barbosa Lima, Martin Lindsey Christoffersen

**Affiliations:** 1 Laboratório de Bentos Costeiro, Departamento de Biologia, Universidade Federal de Sergipe, São Cristóvão, Sergipe 49100-000, Brazil; 2 Departamento de Sistemática e Ecologia, Universidade Federal da Paraíba, Campus I, Castelo Branco, João Pessoa, Paraíba 58051-900, Brazil

**Keywords:** Micromollusks, Caenogastropoda, Caecinae, Western Atlantic, South America, continental shelf, shallow waters

## Abstract

After an extensive search for the type specimens of *Caecum floridanum* Stimpson, 1851, we believe that these specimens may have been either lost or destroyed in the Chicago fire (1871). This paper presents a redescription of the species and a neotype is designated based on material from the type locality (Florida). Protoconch and growth stages of *Caecum floridanum* are described and illustrated herein. The teleoconch IV of *Caecum floridanum* is characterized by strong, wide, low, rounded, closely arranged axial ribs, except last three to four preceding the aperture, which are larger and more widely separated. *Caecum compactum* Dall, 1892 is here synonymized under *Caecum floridanum*. *Caecum annulatum* Emmons, 1858 and *Caecum dux* Folin, 1871 are not considered synonyms of *Caecum floridanum* in this report.

## Introduction

Stimpson (1851: 112) described the marine gastropod *Caecum floridanum* from specimens collected on the coast of Florida (USA). Stimpson’s description for this species is brief, with no illustration and no information on the type material, depository institution(s) or habitat.

According to Dance (1966: 302), shells studied by Stimpson were deposited in the Chicago Natural History Museum (CNHM), currently called the Field Museum of Natural History (FMNH), Illinois, Chicago, USA, and destroyed in the Chicago fire (1871). However, the institution destroyed was the Chicago Academy of Sciences (CAS), where Stimpson had stored the malacological material studied (Hendrickson and Beecher 1972). According to Bartsch et al. (1946: 10) and Warén (1980), types described by Stimpson were deposited in the “J.G. Jeffreys” collection and Zoological Museum of the University of Copenhagen (ZMUC), respectively. However, Cernohorsky (1974) and Dr Ole S. Tendal (Curator of Mollusca – personal communication, June 2008) found no specimens of *Caecum floridanum* in the ZMUC collection. Moreover, a number of years after Jeffreys death, his conchological collection was given by Dall to the U.S. National Museum of Natural History (USNM, Smithsonian Institution) (Dance 1966: 289–290, Warén 1980: 3). Some years later, a part of the material collected during the Lightning, Porcupine and Triton expeditions was given to BMNH (actually NHMUK) (Warén 1980: 4). However, based on information from the respective curators, no type material for *Caecum floridanum* was found in either institution. Thus, we conclude that all types of this species were deposited in the CAS and lost or destroyed in the Chicago fire.

*Caecum floridanum* is a shallow water species widespread throughout the Western Atlantic and associated with a variety of ecosystems and biotopes (Abbott 1974, Vokes and Vokes 1983, Leal 1991, Lightfoot 1992, Diaz and Puyana 1994, Bandel 1996, Rios 2009, Tunnell et al. 2010, Redfern 2013, Lima et al. 2015).

The present study provides a detailed re-description for *Caecum floridanum* based on a large number of specimens studied from the Western Atlantic and the designation of a neotype for the species based on a specimen from the type locality (Florida). In addition, the protoconch and all growth stages of this species are described and figured here based on scanning electron microscopy.

## Materials and methods

Identification of the material was performed under a stereomicroscope. Specimens were also studied based on photographs taken with scanning electron microscopy (SEM), at the Electron Microscope Laboratory of the “Museu Nacional do Rio de Janeiro (MNRJ)”.

Growth stages in shells were recognized based on truncation regions characterized herein as strangulation (Fig. 2C), suture (Fig. 2G), pronounced increase in diameter (Fig. 3A), or with an interface of sculpture patterns (Figs 2A–B to 2C–E, 3–4). Roman numerals discriminate and arrows delimit each growth stage. Some growth stages were characterized together (e.g., Fig. 2D: II–III) due to the lack of a distinct truncation region [see approach originally proposed in Lima et al. (2013)].

**Figure 1.**
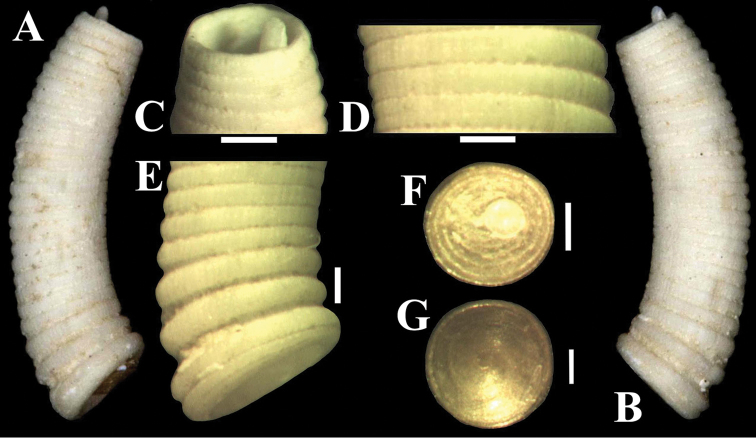
Photos of *Caecum floridanum*, teleoconch IV (neotype, ANSP 407671): **A–B** lateral view **C** Apical region showing mucro **D** Detail of longitudinal lines and axial interspaces/ribs **E** Anterior region view **F** Operculum (outer surface view) **G** Operculum (internal surface view). Scale bars: 500 μm (**A–B**), 200 μm (**C, E**), 100 μm (**D, F–G**).

**Figure 2.**
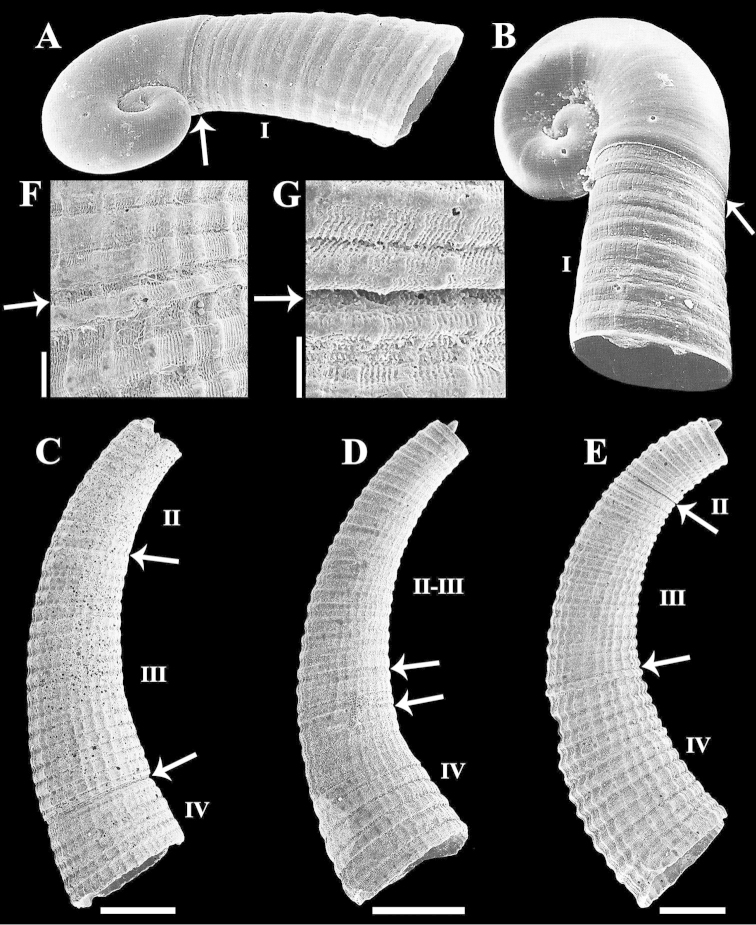
Scanning electron micrographs of Caecum floridanum shells at different growth stages: **A–B** Protoconch and teleoconch I (Bandel 1996: pl. 7, fig. 8, 0.3 mm, fig. 7, 0.7 mm, respectively) **C** Teleoconch II to IV (IBUFRJ 12687) **D** Teleoconch II to IV (IBUFRJ 12689) **E** Teleoconch II to IV (MORG 41.867) **F** Truncation region between teleoconch II and III **G** Truncation region between teleoconch II and III. Scale bars: 500 μm (**C–E**), 100 μm (**F**), 50 μm (**G**).

**Figure 3.**
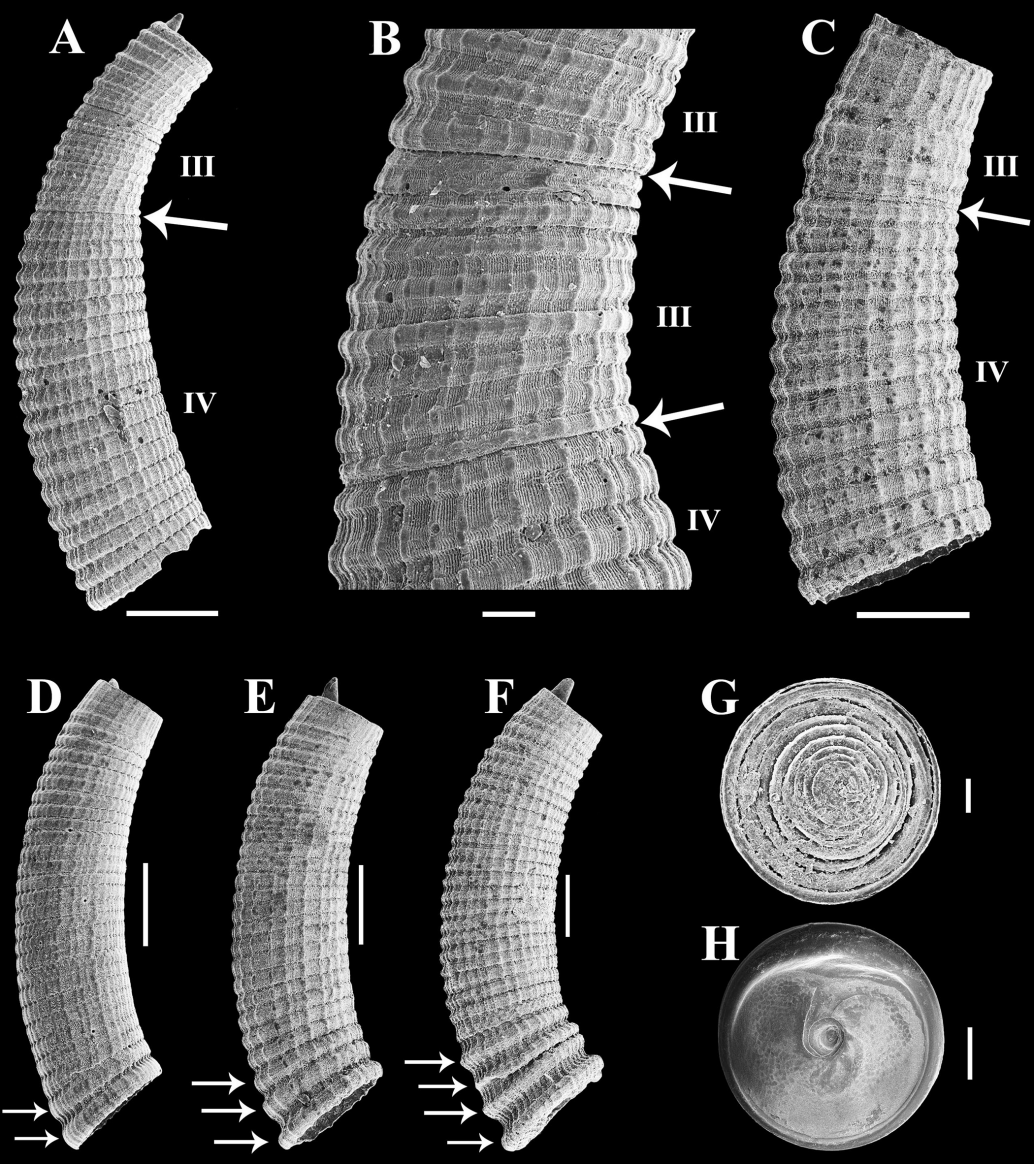
Scanning electron micrographs of *Caecum floridanum* shells at different growth stages and operculum: **A** Teleoconch II to III (MORG 41.867) **B** Truncation region between teleoconch II and III (**A**) **C** Teleoconch II to III (IBUFRJ 18376) **D–F** Teleoconch IV (**E–F** Arrows pointing to last three to four axial ribs at anterior end) (**D–F** IBUFRJ 18376) **G** Operculum, outer surface (IBUFRJ 7408) **H** Operculum, inner surface (IBUFRJ 7408). Scale bars: 500 µm (**A, C, E–H**), 100 µm (**B, I**), 200 µm (**D**).

**Figure 4.**
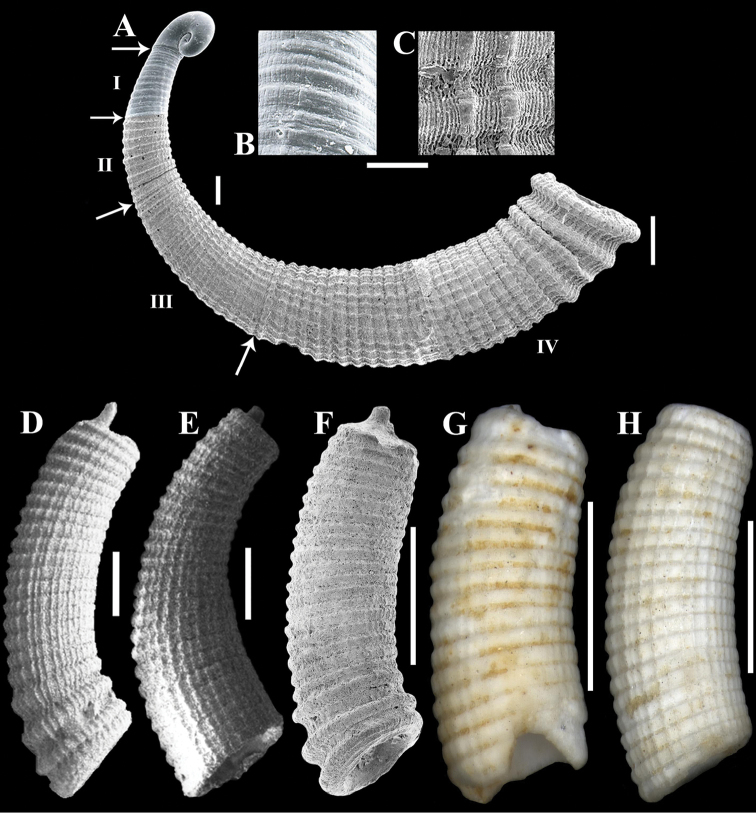
Hypothesis in the reconstruction of growth stages and synonyms of *Caecum floridanum*: **A** Protoconch to teleoconch IV **B** Sculpture of teleoconch I **C** Sculpture of teleoconch II to IV **D** *Caecum irregular*, teleoconch IV (syntype – MNHN 25729) **E** *Caecum phronimum* at different growth stages, teleoconch II to III (syntype – MNHN 25728) **F** *Caecum compactum*, teleoconch IV (USNM 83590) **G–H** *Caecum puntagordanum* (holotype – PRI 26107 and paratype – PRI 26108, respectively), teleoconch IV. Measures and scale bars: **A** protoconch and first half of teleoconch I (Bandel 1996: pl. 7, fig. 7, 0.7 mm), second half of teleoconch II to III (200 µm), teleoconch IV (500 µm), 100 µm (**B–C**), 500 µm (**D–E**), 1 mm (**F–H**)

The following standard measures are based on Lima et al. (2013) and were taken using a stereomicroscope with an eyepiece micrometer: total length (Tol), length from the aperture to the point of maximum arc (Larc), maximum arc (Arc), diameter of aperture (Da), diameter of posterior extremity (Dpe), length of mucro (Lm) and width of mucro (Wm). Only undamaged shells were measured. Simple descriptive statistics were performed to determine the range of meristic and morphometric variables. Other abbreviations used: number (N), mean (M), range (R), standard deviation (SD). The number inside brackets indicates the number of specimens in each lot.

Part of the material examined was obtained from the following projects organized by Brazilian Government: Estudo Multidisciplinar da Plataforma Continental da Amazônia (AMASSEDS/Brazil: 1970/1979); “Geologia Marinha da Plataforma Continental do Brasil” (GEOMAR: 1989-1990/1997, Brazil); “Programa de Avaliação do Potencial Sustentável dos Recursos Vivos da Zona Econômica Exclusiva” (REVIZEE/Brazil).

Most of specimens analyzed was obtained on loan and are deposited in the following scientific collections: ANSP – Academy of Natural Sciences of Philadelphia, Philadelphia, USA; IBUFRJ – Instituto de Biologia, Universidade Federal do Rio de Janeiro, Rio de Janeiro, Brazil; LMUFRPE – Laboratório de Malacologia, Departamento de Pesca e Aquicultura, Universidade Federal Rural de Pernambuco, Recife, Pernambuco, Brazil; MNHN – Muséum national d’Histoire naturelle, Paris, France; MNRJ – Museu Nacional, Universidade Federal do Rio de Janeiro, Rio de Janeiro, Brazil; MORG – Museu Oceanográfico Prof. Eliezer de Carvalho Rios, Fundação Universidade Federal do Rio Grande, Rio Grande, Rio Grande do Sul, Brazil; MZSP – Museu de Zoologia, Universidade de São Paulo, São Paulo, Brazil; PRI – Paleontological Research Institution, New York, USA; UF – Florida Museum of Natural History, University of Florida, Gainesville, Florida, USA; UFPB MOLL – Laboratório de Invertebrados Paulo Young, Departamento de Sistemática e Ecologia, Universidade Federal da Paraíba, João Pessoa, Paraíba, Brazil.

## Systematics

### Caecidae Gray, 1850
Caecinae Gray, 1850

#### Caecum

Taxon classificationAnimaliaLittorinimorphaCaecidae

Fleming, 1813

##### Type species.

*Dentalium trachea* Montagu, 1803 (by subsequent designation, Gray 1847: 203) from the Atlantic coasts of Europe, the Mediterranean Sea and northwestern Africa (Vannozzi et al. 2015).

#### Caecum floridanum

Taxon classificationAnimaliaLittorinimorphaCaecidae

Stimpson, 1851

Figs 1 , 2 , 3 , 4

Caecum floridanum Stimpson 1851: 112 (Recent, Florida).
Caecum irregulare Folin 1867: 47, pl. 3, fig. 6 (Bahia state, northeastern Brazil; syntype MNHN 25729; Recent) – Dall (1892: 298), Abbott (1974: 92, fig. 874), Rios (1975: 40, pl. 11, fig. 142, 1985: 44, pl. 17, fig. 199, 1994: 57, pl. 18, fig. 211, 2009: 98, fig. 237), Diaz and Puyana (1994: 141, pl. XLV, fig. 489), Redfern (2001: 41, pl. 20, fig. 174a–b) [Fig. 4D, type material].
Caecum phronimum Folin 1867: 44, pl. 3, fig. 4 (Port au Prince, Haiti; syntype MNHN 25728; Recent) – Abbott (1974: 92, fig. 874), Rios (1985: 44, pl. 17, fig. 199, 1994: 57), Diaz and Puyana (1994: 141, pl. XLV, fig. 489), Absalão and Pizzini (2002: pl. 1, fig. 2, pl. 2, Figs 15–16) [Fig. 4E, type material].
Caecum floridanum var. compactumDall 1892: 298, pl. 20, fig. 9b (Caloosahatchie River, near Fort Thompson, Florida; type USNM 83590; fossil, Pliocene) – new synonym [Fig. 4F, type material].
Caecum cayoense Rehder 1943: 190, pl. 20, fig. 9 (Bonefish Key, Florida Keys; holotype USNM 536045; Recent) – Abbott (1974: 92, fig. 874), Tunnell et al. (2010: 144).
Caecum puntagordanum Weisbord 1962: 165, pl. 14, Figs 13–14 (south flank of Punta Gorda anticline, Venezuela; holotype PRI 26107, paratype, PRI 26108; fossil, Pliocene] – Abbott (1974: 92, fig. 874), Tunnell et al. (2010: 144) [Fig. 4G–H, type material].

##### Type material.

NEOTYPE ANSP 407671 (herein designated – Fig. 1), USA, Florida: Venice – collected by Donald R. Moore, June 1963.

##### Additional material examined.

United States of America: -- off Florida State: [8] ANSP 100196, Bahia Honda Key; [1] ANSP 141044, Dry Tortugas, 1925, 29 m; [13] ANSP 306229, John’s Pass, 1965, 62 m; [1] UF 350743, Palm Beach, 01 April 1979; [1] UF 359106, Crawl Key, 01 August 1978; [1] UF 359111, Cayo Costa, beach drift, 01 April 1992; [11] UF 359112, Palm Beach, beach drift, 01 February 1988; [1] IBUFRJ 1920, collector Tarrasconi, subtidal, 04 February 1999; [5] MZSP 42358, [6] MZSP 91154, collector P.J. Souza, Deerfield Beach, 3 to 5 m, January 1999; Bahamas: [3] UF 359107, Cat Key, beach drift, 01 March 1981; [3] UF 359108, Sampson Cay, beach drift, 01 June 1992; West Indies: -- off Virgin Islands: [1] UF 359109, St. Croix, 19.81 m, 01 January 1993; -- off ABC Islands: -- Aruba Island: [4] IBUFRJ 6500, [11] IBUFRJ 6906, collector F. Verberne; -- off Venezuela: [1] PRI 26107, holotype and [1] PRI 26108, paratype (*Caecum puntagordanum*), Tertiary, Lower Pliocene, Mare Formation, Punta Gorda Anticline; -- off Trinidad and Tobago: [6] UF 359105, Tobago Island, 1.82 to 8.53 m, 01 January 1992; [14] UF 359113, Tobago Island, beach drift, 01 April 1991; Brazil: -- off Amapá State: AMASSEDS, collector R/V ‘Columbus Iselin’ – [5] MORG 39.824, April 1997; [8] MORG 43.297, station 4134, 45 to 50 m, March 1997; -- off Pará State: GEOMAR, collector R/V ‘Almirante Saldanha’ – [2] MORG 15.815, Cânion do Amazonas, station 2438, 40 m, 1970; [5] MORG 15.902, Rio Pará, 25 m, 1970; [2] MORG 16.517, Foz do Amazonas, station 2438, 40 m, 08 November 1970; AMASSEDS, collector R/V ‘Columbus Iselin’, cruise III – [3] IBUFRJ 18306, station 3209, 01°21'N, 48°00'W, 53 m, May 1990; [2] IBUFRJ 18308, station 3210, 01°52'N, 48°16'W, 47 m, 12 May 1990; [1] IBUFRJ 18309, station 3228, 03°25'N, 49°55'W, 74 m, 17 May 1990; [3] IBUFRJ 18310, station 3201, 00°29'N, 48°11'W, 12 m, May 1990; [1] IBUFRJ 18377, station 3210, 01°52.45'N; 48°16'W, 47 m, 05 December 1990; -- off Maranhão State: REVIZEE/Score NORTE, collector Márcia – [15] IBUFRJ 18316, Banco do Tarol, 20 July 1997; -- off Rio Grande do Norte State: collector MORG research group – [dozens] MORG 19.119, [7] MORG 26.453/28.186, Atol das Rocas, February 1977; -- off Paraíba State: [2] MZSP 77776, Formosa beach, Cabedelo, January 1979, collector L.R.L. Simone; [12] UFPB MOLL 3545, [02] MZSP 114729, Cabo Branco Beach, in rhodolith beds, 22 December 2011, collector André, Emerson, Jéssica, Lívia, Rafael and Silvio; -- off Pernambuco State: [4] IBUFRJ 11179, Rata Island, Fernando de Noronha Archipelago, 08 August 1999, collector IBUFRJ research group; [3] MORG 32.949, Fernando de Noronha Archipelago, 40 m, 05 December 1986, collector M. Cabeda; [3] MZSP 32004, Fernando de Noronha Archipelago, 0–6 m, 20 July 1999, collector P.J. Souza and L.R.L. Simone; collector LMUFRPE research group – [3] LMUFRPE, Porto de Galinhas beach, 05 October 1982; [3] LMUFRPE, Suape, 24 May 1982; -- off Alagoas State: [2] MORG 12.494, Rec. da Marinha, 1964, collector Sá Cardoso; -- off Bahia State: [9] IBUFRJ 7408, [2] IBUFRJ 7287, Ribeira, Salvador, 1994, collector L. Trinchão; [3] MORG 41.867, Recôncavo Baiano, 29 April 1997, collector ‘fishing-boat’; [1] MORG 45.602, Boipeba, 45 m, December 2002, collector R/V ‘Astro Garoupa’; [3] MORG 45.639, Camamu Bay, 52 m, 11 December 2002, collector R/V ‘Astro Garoupa’; [5] MZSP 44883, Coroa Vermelha Reef, Salvador, 13 January 2000, collector E.P. Gonçalves and P.J. Souza; [7] MORG 18.052, Abrolhos Archipelago, 5 m, July 1972, collector L.C. Araújo; collector MORG research group - [23] MORG 20.113, Abrolhos Archipelago, February 1978; [27] MORG 23.836, Abrolhos Archipelago, January 1985; [29] MORG 26.418, I. Guarita, Abrolhos Archipelago, 5 m, February 1987, collector A.S.J.L. Laurino; [1] MZSP 36863, Alcobaça, Parcel Paredes, 2–3 m, 16 January 2000, collector E.P. Gonçalves and P.J.S. Souza; REVIZEE/Score Central, collector R/V ‘Antares’ – [132] IBUFRJ 10134, [4] IBUFRJ 12679, [10] IBUFRJ 12750, station C76, 15°54'22"S, 38°31'09"W, 66 m, 30 April 1996; [9] IBUFRJ 14688, station 2R, 13°38'S, 38°44'W, 55 m, 02 July 2001; [3] IBUFRJ 18307, [7] IBUFRJ 18315, [4] IBUFRJ 18376, [2] IBUFRJ 18378, [1] IBUFRJ 18379, station R4#1, 13°45'S, 38°23'W, 91 m, 23 June 2002; [5] IBUFRJ 18313, station R3#1, 15°49'S; 38°36'W, 83 m, 21 June 2002; local project – [6] MNRJ 14061, 13°29'22"S, 38°48'43"W, vi.2007; [5] MNRJ 14062, 13°28'17"S, 38°48'44"W, August 2007; [2] MNRJ 14069, 13°29'20"S, 38°47'37"W, August 2007; [3] MNRJ 14071, 13°28'17"S, 38°48'44"W, August 2007; [1] MNRJ 14073, 13°29'20"S, 38°47'37"W, August 2007; [1] MNRJ 14076, 13°16'00"S, 38°55'07"W, 12 January 2007; [6] MNRJ 14081, 13°19'51"S, 38°52'51"W, 12 January 2007; [1] MNRJ 14090, 13°28'58"S, 38°49'06"W, August 2007; [3] MNRJ 14092, 13°28'58"S, 38°48'21"W, August 2007; -- off Espírito Santo State: [1] IBUFRJ 8629, Piúma, 1993, collector IBUFRJ research group; GEOMAR XII, collector R/V ‘Almirante Câmara’ - [7] IBUFRJ 7289, 20°53'S, 40°12'W, 26 August 1979; REVIZEE/Score Central, collector R/V ‘Antares’ – [2] IBUFRJ 9280, station C63, 19°40'42"S, 38°08'15"W, 61 m, 25 April 1996; [30] IBUFRJ 9421, [2] IBUFRJ 12752, station C65, 18°53'37"S, 39°06'23"W, 50 m, 25 April 1996; [30] IBUFRJ 9817, [31] IBUFRJ 12689, station C62, 20°30'02"S, 37°28'51"W, 96 m, 25 April 1996; [8] IBUFRJ 10841, station VV31, 18°52’ S, 39°35'W, 23 m, 28 February 1996; [4] IBUFRJ 11360, station VV22, 20°20'S, 40°15'W, 33 m, 27 February 1996; [4] IBUFRJ 12681, station VV21, 20°38'S, 40°00'W, 56 m, 27 February 1996; [2] IBUFRJ 12687, station VV16; 21°10'S, 40°27'W, 28 m, 26 February 1996; [2] IBUFRJ 14574, station 42R, 20°44'S, 31°49'W, 85 m, 11 July 2001; [10] IBUFRJ 18311, station Y7, 20°50'S, 40°10'W, 75 m, 28 June 2002; [4] IBUFRJ 18314, station VV22, 20°20'S; 40°59'W, 33 m, 27 February 1996; [6] MORG 40.457, station VV31, 18°52'S, 39°35'W, 23 m, 28 February 1996); [5] MORG 41.084, station VV21, 20°38'S, 40°00'W, 56 m, 27 February 1996; [17] MORG 33.637, Trindade and Martim Vaz Archipelago, 75 m, 25 April 1996; [3] MORG 39.124, 18°53'S, 39°06'W, 50 m, 25 April 1996; -- off Rio de Janeiro State: [2] MZSP 63394, Rio das Ostras, September 1971, collector MZSP research group; GEOMAR XII, collector R/V ‘Almirante Câmara’ - [2] IBUFRJ 7288, 22°05'S, 40°17'W, 29 August 1979.

##### Original description.

“Shell much arcuated, somewhat thick, white, shining; with about thirty-two sharp, elevated ribs, much narrower than their interspaces. Aperture slightly oblique, not contracted. In some specimens there is a broad rib just above the aperture. Long. .075; lat. .02. poll. *Hab.* Florida.” (Stimpson 1851: 112).

##### Diagnosis.

Teleoconch with strong, wide, low, rounded, closely arranged axial ribs, except last three to four preceding the aperture, which are larger and more widely separated.

##### Redescription

**(shell – neotype).** Teleoconch IV (last growth stage) small (Tol 3.85 mm), tubular, rather thick, moderately and regularly arched (Larc 1 mm; Arc 0.30 mm), with slight increase in caliber from apical region to aperture, opaque-white to cream–white with brownish markings (Fig. 1A–B). Surface sculptured with longitudinal striae, faint to well-defined longitudinal threads (Fig. 1D) and 26 prominent, wide, low, rounded, closely arranged and regularly spaced axial ribs (Fig. 1A–B), except last three preceding aperture, which are larger and more widely separated (Fig. 1E). Striae and threads cross ribs and interspaces (Fig. 1D); threads producing a very slightly beaded effect on ribs (Fig. 1D). Axial interspaces very narrow and shallow, except last two to three preceding aperture, which become wider and deeper (Fig. 1E). Apical region circular (Dpe: 0.57 mm) (Fig. 1C). Septum slightly convex, deeply recessive (Fig. 1C). Mucro finger-shaped, conical, moderately slender (Lm: 0.12 mm; Wm: 0.15 mm), positioned on dorsal margin, straight (Fig. 1C). Aperture circular (Da 0.75 mm), prominent varix around (Fig. 1A–B, E). Operculum yellowish-brown, horny; outer surface concave, with nucleus subcentral, about eight slight coil (Fig. 1F); inner surface convex, smooth (Fig. 1G).

##### Characterization.

**Protoconch to teleoconch IV.** Protoconch paucispiral (about 1.5 whorls), planispiral, smooth; suture deep, grooved; transition to teleoconch I abrupt, marked by slight axial edge (Figs 2A–B, 4A). Teleoconch I short, sculptured with 9 to 15 wide, very weak, slightly wavy, closely arranged axial riblets and very fine, slight longitudinal striae (Figs 2A–B, 4A); transition to teleoconch II not observed. Teleoconch II sculptured with 9 to 15 faintly demarcated, well-spaced axial riblets and very weak longitudinal threads and striae (Figs 2C–E, 4A); transition to teleoconch III not clear or marked by very slight axial strangulation/suture (Fig. 2F–G). Teleoconch III to IV sculptured with wide, rounded, low, closely arranged axial ribs, longitudinal striae and threads that increase in prominence with the progression of stages (Figs 2C–E, 3A–C). Teleoconch III with about 18 axial ribs (Fig. 2C–E); transition to teleoconch IV not clear (Figs 2E, C) or marked by very slight axial strangulation to pronounced increase in diameter (Figs 2C–D, 3A–B). Teleoconch IV small (Tol 2.90–4.25 mm, M 3.53 mm, N 50), arched (Larc 0.85–1.50 mm, M 1.11 mm, N 50; Arc 0.20–0.40 mm, M 0.28 mm, N 50), apical region circular (Dpe 0.37–0.57 mm, M 0.45 mm, N 50), mucro finger-shaped to triangular, conical (Lm 0.07–0.25 mm, M 0.15 mm, N 49; Wm 0.07–0.20 mm, M 0.13 mm, N 50), aperture circular (Da 0.50–0.75 mm, M 0.58 mm, N 50), sculptured with 22 to 33 axial ribs, wider in comparison to previous stages (Figs 2C–E, 3A–F, 4A), last three to four usually larger and more separated (Figs 1E, 3D–F, 4A, D, F). Figure 4A shows the reconstruction of the growth stages.

##### Type locality.

Florida (Venice), United States (here established).

##### Geographic distribution.

North Carolina to Florida (Dall 1892, Rehder 1943, Olsson and Harbison 1953, Abbott 1974, Gomes and Absalão 1996); Texas (Tunnell et al. 2010); Mexico (Vokes and Vokes 1983, Lightfoot 1992); Bahamas (Kisch 1959, Redfern 2001); Cuba (Espinosa et al. 1995); Puerto Rico (Rosenberg 2009); Haiti (Folin 1867); Virgin Islands and Saint Martin (Kisch 1959); Trinidad and Tobago Archipelago (Lightfoot 1992); ABC Islands (Jong and Coomans 1988, Gomes and Absalão 1996); Costa Rica (Sevilla et al. 2003); Panama (Olsson and McGinty 1958, Sevilla et al. 2003); Colombia (Diaz and Puyana 1994, Bandel 1996); Venezuela (Weisbord 1962, Rios 2009); Guiana (Princz 1977); Surinam (Rosenberg 2009); Brazil: Amapá, Pará, Maranhão, Ceará, Rio Grande do Norte, Paraíba (presente study), Pernambuco, Alagoas, Bahia, Espírito Santo (Folin 1867, Dall 1892, Kisch 1959, Leal 1991, Gomes and Absalão 1996, Rios 2009), Rio de Janeiro (present study).

## Discussion

The brief original description (without illustration) and the loss of the types does not permit recognition of the morphotype originally proposed for *Caecum floridanum*. These issues are more than sufficient to make the taxon a *nomen dubium*. However, since 1892 a typical morphotype, which is not in agreement with the conchological characters described by Stimpson (1851) (see also Jong and Coomans 1988: 35, *Caecum irregulare*) has been universally accepted for *Caecum floridanum* in the vast majority of taxonomic and ecological papers. Although the original description is brief, we can recognize that there are considerable discrepancies between the morphotype of the original description and that universally accepted for *Caecum floridanum*. Stimpson described this species as having “thirty-two sharp elevated ribs much narrower than the interspaces”, while the most papers recognize that the taxon has 22 to 33 low, rounded, closely arranged axial ribs and very narrow and shallow axial interspaces, except the last one preceding the aperture. Dall (1892: 298) was the first to characterize this species in disagreement with the original proposition based on *Caecum irregulare* Folin, 1867 (Fig. 4D), which was included as a synonym in the author’s study, without, however, giving any reasons for such an action. Thereafter, a new concept of *Caecum floridanum* *sensu* Dall was established and followed by practically all authors addressing the taxon (Rehder 1943, Olsson and Harbison 1953, Olsson and McGinty 1958, Moore 1970: fig. 2, Abbott 1974, Vokes and Vokes 1983, Leal 1991, Lightfoot 1992, Diaz and Puyana 1994, Bandel 1996, Gomes and Absalão 1996, Lee 2009, Rios 2009, Tunnell et al. 2010, Redfern 2013, Lima et al. 2015). *Caecum floridanum* cannot be identified accurately based on Stimpson’s description, which is too vague and might be applied to various *Caecum* taxa from the Western Atlantic. Therefore, any nomenclature decision regarding this taxon (e.g., description of the taxon as a new species or validating its synonym *Caecum irregulare*, making *Caecum floridanum* a *nomen dubium*) will cause instability, inconsistency and taxonomic confusion (unless some type material is found).

Thus, we believe that the best course is to designate a neotype for *Caecum floridanum* based on a specimen deposited at the ANSP (International Commission on Zoological Nomenclature 1999: art. 75.3.7.) and collected from the type locality (ICZN 1999: art. 76.3.) due to the rather vague original description (in our view, an exceptional need before the ICZN 1999: art. 75.3.). This neotype replaces the lost or destroyed original type material (ICZN 1999: art. 75.3.4, see Introduction to review the steps taken to trace the type material) and clarifies inconsistencies between the concepts put forth by Stimpson (1851) and subsequent authors (ICZN 1999: art. 75.3.1.), conserving the current usage of the name and the universally accepted morphological concept of the species (as have been used in most of the literature) beyond doubt (ICZN 1999: art. 75.3.5.). Vokes and Vokes (1983: 120, fig. 12) recognized a hypotype for *Caecum floridanum*, but this nomenclatural type does not appear in the ICZN (1999) and has no scientific value.

The characterization of teleoconch II presented herein for *Caecum floridanum* is consistent with that of Lightfoot (1992: 179). Bandel (1996) recognized four to five growth stages in the ontogeny of this species, but did not describe each stage separately. Thus, reconstruction of the stages presented by him is an assumption not supported with clear data. Still according to Bandel (1996), a varix is seen on the penultimate and last growth phases, but it is characterized here only at the end of the last stage.

*Caecum floridanum* has been mistakenly figured as *Caecum imbricatum* Carpenter, 1858 by Rios (1994: pl. 19, fig. 212, 2009: 99, fig. 238) and Bandel (1996: fig. 13, pl. 7, Figs 5–8). *Caecum annulatum* Emmons, 1858 and *Caecum dux* Folin, 1871 have usually been considered synonyms of *Caecum floridanum* (Dall 1892, Pilsbry and Aguayo 1933, Rosenberg 2009). A reassessment of the shell morphology of *Caecum annulatum* based on Emmons (1858: 183, fig. 190) and of *Caecum dux* from photos of type material (MNHN), allow us to conclude that both species have somewhat different conchological characters, when compared to *Caecum floridanum*. *Caecum annulatum* has an inflated, dome-shaped septum and rounded, raised, axial ribs, which are not slightly larger and more widely separated preceding aperture (Emmons 1858: 183, fig. 190), while *Caecum dux* has a broad, blunt mucro, raised, widely separated axial ribs and no evidence of longitudinal sculpture on the teleoconch. Two type specimens of *Caecum floridanum* var. *compactum* were recognized by Dall (1892), but at least five shells are deposited in USNM (83590). Only two of these specimens represent *Caecum floridanum* (Fig. 4F). The most distinguishing features of *Caecum floridanum* are the recessive septum, rather triangular mucro, longitudinal striae and threads cross axial ribs and interspaces, aperture with prominent varix and low, rounded, closely arranged axial ribs, except last preceding aperture, which become larger and wider (ICZN 1999: art. 75.3.2.).

## Supplementary Material

XML Treatment for Caecum

XML Treatment for Caecum floridanum
